# Thermal noise limit for ultra-high vacuum noncontact atomic force microscopy

**DOI:** 10.3762/bjnano.4.4

**Published:** 2013-01-17

**Authors:** Jannis Lübbe, Matthias Temmen, Sebastian Rode, Philipp Rahe, Angelika Kühnle, Michael Reichling

**Affiliations:** 1 Fachbereich Physik, Universität Osnabrück, Barbarastraße 7, 49076 Osnabrück, Germany; 2 Institut für Physikalische Chemie, Johannes Gutenberg-Universität Mainz, Duesbergweg 10-14, 55099 Mainz, Germany; 3 now at: SmarAct GmbH, Schütte-Lanz-Strasse 9, 26135 Oldenburg, Germany; 4 now at: Department of Physics and Astronomy, The University of Utah, 115 South 1400 East, Salt Lake City, UT 84112, USA

**Keywords:** Cantilever, feedback loop, filter, noncontact atomic force microscopy (NC-AFM), noise

## Abstract

The noise of the frequency-shift signal Δ*f* in noncontact atomic force microscopy (NC-AFM) consists of cantilever thermal noise, tip–surface-interaction noise and instrumental noise from the detection and signal processing systems. We investigate how the displacement-noise spectral density *d^z^* at the input of the frequency demodulator propagates to the frequency-shift-noise spectral density *d*^Δ^*^f^* at the demodulator output in dependence of cantilever properties and settings of the signal processing electronics in the limit of a negligible tip–surface interaction and a measurement under ultrahigh-vacuum conditions. For a quantification of the noise figures, we calibrate the cantilever displacement signal and determine the transfer function of the signal-processing electronics. From the transfer function and the measured *d^z^*, we predict *d*^Δ^*^f^* for specific filter settings, a given level of detection-system noise spectral density *d^z^*_ds_ and the cantilever-thermal-noise spectral density *d^z^*_th_. We find an excellent agreement between the calculated and measured values for *d*^Δ^*^f^*. Furthermore, we demonstrate that thermal noise in *d*^Δ^*^f^*, defining the ultimate limit in NC-AFM signal detection, can be kept low by a proper choice of the cantilever whereby its *Q*-factor should be given most attention. A system with a low-noise signal detection and a suitable cantilever, operated with appropriate filter and feedback-loop settings allows room temperature NC-AFM measurements at a low thermal-noise limit with a significant bandwidth.

## Introduction

**Figure 1:**
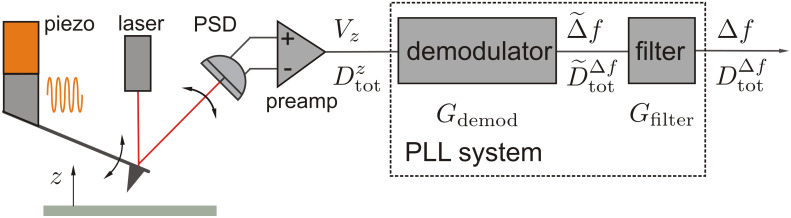
Schematic representation of the signal path in an NC-AFM system based on optical beam deflection with frequency demodulation using a PLL. The amplitude response of the PLL *G*_PLL_ = *G*_filter_ × *G*_demod_ is split into demodulation and filtering parts, which are described by *G*_demod_ and *G*_filter_. The quantities *V_z_* and Δ*f* describe the input and output signals of the PLL in NC-AFM operation while 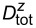 and 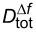 are the corresponding noise power spectral densities superimposed to the signals.

In this contribution, we discuss noise in frequency-modulation noncontact atomic force microscopy (NC-AFM) using cantilevers as force sensors and optical beam deflection (OBD) for signal detection. Figure 1 shows a schematic diagram of an NC-AFM setup based on OBD to illustrate the signal path and the quantities describing noise. Measured quantities discussed here are often electrical signals that are equivalent to quantities describing the mechanical oscillation of the cantilever. The calibration procedure described in Section 1 of Supporting Information File 1 establishes a relation between the representation in mechanical and electrical units. During NC-AFM operation, the cantilever with eigenfrequency *f*_0_ is excited to oscillation at the resonance frequency *f_r_*, which differs from its eigenfrequency by the frequency shift Δ*f* = *f_r_* − *f*_0_ when there is a tip–surface interaction. The mechanical oscillation, i.e., a periodic displacement *z*(*t*) of the cantilever with amplitude *A*, is converted into the oscillation signal *V_z_*(*t*) by the position-sensitive detector (PSD) connected to the preamplifier. The amplitude *A* of this signal is determined and stabilised to a preset value by the amplitude feedback loop. Signal processing in NC-AFM involves the demodulation of the periodic cantilever-displacement signal *V_z_*(*t*) as well as filtering in the frequency domain to yield the frequency shift Δ*f*(*t*) carrying the information on the tip–surface interaction [1]. Demodulation is commonly performed by a phase-locked loop (PLL) circuit [2]. As schematically depicted in Figure 1, the amplitude response of the PLL unit can formally be decomposed into the amplitude response *G*_demod_ of the demodulator and the amplitude response *G*_filter_ of an in-loop or output filter. The characteristics of *G*_filter_ can be set by the user according to the needs of the experiment.

Noise in NC-AFM consists mainly of three contributions: noise arising from the thermal excitation of a cantilever or another force sensor, noise caused by the detection system and signal processing electronics [3–4], and instabilities arising from the interaction of the force microscopy tip with the surface as well as arising from the feedback loops stabilising the cantilever oscillation amplitude and the tip–surface distance [5]. Here, we investigate noise for the case of negligible tip–surface interaction and discuss the cantilever-displacement thermal-noise spectral density 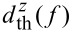 as well as the displacement-equivalent noise spectral density 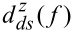 introduced by the detection system. This is carried out here in search of the ultimate limits of detection defined by thermal noise, while a systematic study of the tip–sample interaction noise that is present in any NC-AFM imaging or spectroscopy experiment will be the subject of forthcoming work. Here, we entirely focus the discussion on cantilever-based NC-AFM; however, the concepts, theoretical framework, and experimental strategies for the noise analysis can easily be transferred to systems based on other force sensors and detection schemes.

Under ultrahigh-vacuum (UHV) conditions, the thermal noise of the cantilever is usually small compared to the noise of the detection system due to the high *Q*-factor of the cantilever in vacuum [6]. The instrumental noise sources in an optical beam deflection (OBD) setup were recently discussed in detail [3] and it was found that the major noise sources are shot noise arising from the photodetector as well as Johnson noise originating from the resistors in the preamplifier. Further noise is generated in the laser diode that is mainly quantum noise for small output power and mode-hopping noise for large output power [3]. Back reflections of the laser beam into the laser optical resonator may increase mode hopping. The laser spot on the photodiode may further be disturbed by optical interference, creating time-varying speckle patterns due to temperature fluctuations and mechanical instability. It has been shown, however, that by operating the laser diode with radio-frequency modulation, the contribution of the light source to the total noise can be reduced to a negligible minimum [3].

**Figure 2:**
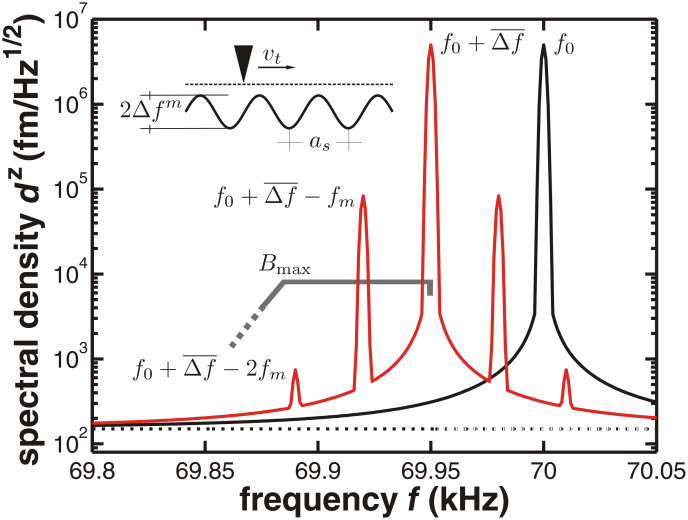
Illustrative representation for the spectral density of the displacement of a cantilever excited to oscillation with 10 nm amplitude at its eigenfrequency *f*_0_ = 70 kHz without tip–sample interaction (black curve) and with tip–sample interaction resulting in a frequency modulation (red curve). Data is drawn for a modulation frequency of *f_m_* = 30 Hz, a modulation amplitude of Δ*f^m^* = 1 Hz and a mean frequency shift of 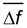 = −50 Hz. A typical detection-system noise floor of 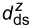 = 150 fm/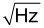 (dotted line) as well as thermal noise based on the cantilever properties (*k* = 2.5 N/m, *Q*_0_ = 100000) are added to the signal. The inset schematically illustrates how scanning the tip over the sample having a spatial periodicity *a_s_* with a scan speed of *v_t_* yields a modulation at frequency *f_m_* = *v_t_*/*a_s_*. The surface corrugation Δ*z* yields a modulation amplitude Δ*f^m^* where the modulation index is Δ*f^m^*/*f_m_* = 1/30 for this example.

The issue of noise is intimately related to the requirements of the NC-AFM system to process signals varying in time. The detection bandwidth *B* needed to retrieve the full information present in the Δ*f*(*t*) signal at the output of the PLL system depends on the spectral components produced during a scanning or spectroscopy experiment. Practically, the maximum usable bandwidth *B*_max_ is defined by the total displacement-noise spectral density 
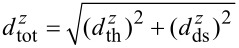
 as schematically illustrated in Figure 2. In this figure, we show the displacement spectral density *d^z^*(*f*) present at the input of the frequency demodulator with contributions of the measurement signal and noise (see Figure 1) as a function of the frequency *f*. This quantity is the root of the one-sided power spectral density *D^z^*(*f*), which is derived from the displacement signal *V_z_*(*t*) via a Fourier transform as


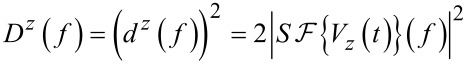


where *S* is the calibration factor converting voltage into displacement as defined in Section 1 of Supporting Information File 1 and 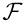 the Fourier transform of the displacement signal *V_z_* with:


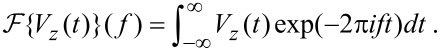


For the case of absent tip–surface interaction, *d^z^* is a sharp peak centred at the cantilever eigenfrequency *f*_0_ (*f*_0_ = 70 kHz in Figure 2) including noise contributions from 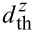 and 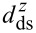, which will be described in detail below. In the presence of a tip–surface interaction, the resonance peak is shifted by the amount 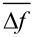 (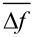 = −50 Hz in Figure 2) caused by the time-invariant part of the interaction. Additionally, sidebands appear that represent spectral components in *V_z_*(*t*) created during scanning or spectroscopy. For simplicity, we assume here a scanning of the tip over the surface with a speed *v_t_* where a periodic corrugation (period *a_s_*) of the surface Δ*f^m^* creates a sinusoidal modulation at the frequency *f_m_* = *v_t_*/*a_s_* (*f_m_* = 30 Hz in Figure 2), i.e., Δ*f*(*t*) = 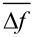 + Δ*f^m^*sin(2π*f_m_* + φ). Effectively, this is a frequency modulation of *V_z_*(*t*) with a modulation index Δ*f^m^*/*f_m_* producing an infinite number of higher harmonics with rapidly decreasing power [4]. How many of these side peaks can be detected depends on the modulation index of the signal and the noise characteristics of the measurement system. For the hypothetical measurement illustrated in Figure 2, only two sideband peaks are well above the noise floor. Here, the suitable bandwidth *B*_max_ is defined by the frequency of the second sideband peak.

The frequency demodulator extracts the frequency shift Δ*f*(*t*) from the periodic displacement signal *V_z_*(*t*) and, for an arbitrary signal, projects the power in the sidebands of *D^z^*(*f*) into the frequency-shift power spectral density *D*^Δ^*^f^*(*f_m_*), which can be represented as:


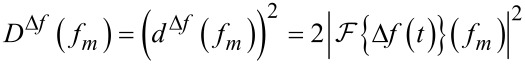



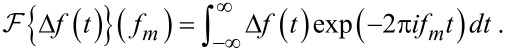


The frequency shift Δ*f*(*t*) varies on a time scale that in an imaging experiment is determined by the spatial periodicity of the scanned structure and the scanning speed, rather than by the period of the cantilever oscillation. Therefore, the spectrum of the frequency shift signal present at the output of the demodulator has significant power only in a limited spectral range of *f_m_*. The detection bandwidth *B* of the demodulator is, therefore, usually restricted to a value of the order of 100 Hz to 1 kHz. As the noise is transformed by the demodulator in a similar way, we define 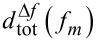 and 
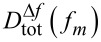
 as the frequency-shift-noise spectral density and the frequency-shift-noise power spectral density, respectively, and discuss separate noise contributions 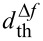 and 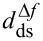 to the frequency-shift signal Δ*f*, as the noise contributions of the thermal cantilever excitation and the detection system yield different spectral characteristics. The detection bandwidth *B* and, consequently, the noise propagation characteristics depend on the PLL amplitude response *G*_PLL_ = *G*_filter_ × *G*_demod_, which can usually be influenced by the operator through the filter settings (see Figure 1).

To understand the influence of various experimental parameters and the settings of the PLL filter on 
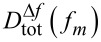
, which is the most relevant noise figure in the NC-AFM experiment, we derive noise models based on system parameters. Hypotheses and conclusions are tested against the reality of NC-AFM experiments, by comparing the noise figures and filter settings for three NC-AFM systems based on the OBD scheme and comparing experimental results to the predicted settings for noise-optimised operation. We find that by the correct choice of the cantilever, by using optimised detection electronics and by appropriate PLL filter settings, the frequency-shift signal Δ*f* can be detected at a low thermal-noise limit over a bandwidth *B* that is more than 100 Hz for room temperature operation under UHV conditions. The dependence of the thermal limit and other noise figures on relevant experimental parameters is discussed in detail.

### Displacement noise

Here, we discuss the displacement noise superimposed on the displacement signal *V_z_*(*t*) in the case of negligible tip–surface interaction. Usually, the signal *V_z_*(*t*) is a noisy sinusoidally oscillating voltage and the noise can be described in the frequency domain by the displacement-noise spectral density 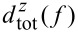. This is the square root of the displacement-noise power spectral density 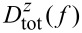, which is proportional to the unwanted energy per frequency interval stored in the oscillating system.

A cantilever that is not deliberately excited but in equilibrium with a thermal bath at temperature *T* exhibits random fluctuations resulting in measurable noise in the cantilever displacement signal. This noise can be predicted by a model outlined in Section 2 of Supporting Information File 1. Furthermore, all electrical and optical components that are part of the detection system produce noise, superimposed on the displacement signal. Therefore, the power spectral density of the total displacement signal noise 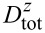 can be described as

[1]
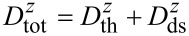


where 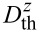 and 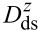 represent the thermal and the detection-system contributions. The quantity 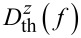 as derived in Section 2 of Supporting Information File 1 can be represented as:

[2]
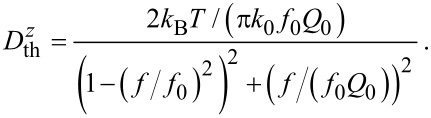


**Figure 3:**
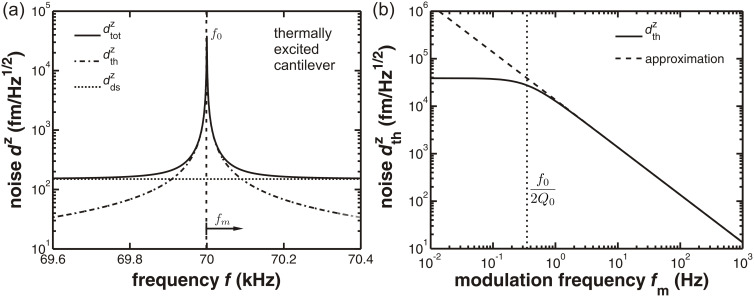
Illustrative representation of noise properties for a cantilever with *f*_0_ = 70 kHz, *k* = 2.5 N/m and *Q*_0_ = 100000 that is solely excited by its contact to a thermal bath at room temperature. (a) Calculated total-displacement noise spectral density 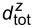 (solid line) compared to the thermal-noise contribution 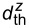 (dash-dotted line) and the detection-system noise 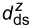 = 150 fm/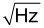 (dotted line). (b) Comparison between the thermal-displacement noise spectral density 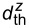
(*f*_0_ ± *f_m_*) as given in Equation 2 (solid line) and the approximation of Equation 3 (dashed line) for the cantilever with a corner frequency of *f*_0_/(2*Q*_0_) = 0.35 Hz. Considering the oscillating cantilever as a mechanical low-pass filter for the displacement noise close to *f*_0_, the corner frequency defines the point at which the noise is attenuated by 3 dB. At modulation frequencies larger than the corner frequency, 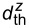 decreases essentially as 1/*f_m_*.

Here, 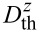 is calculated only for the fundamental cantilever oscillation mode with eigenfrequency *f*_0_, stiffness *k*_0_ and *Q*-factor *Q*_0_ as the contribution of higher harmonics to the total noise power spectral density is small; the fundamental mode contains 97% or more of the total power extracted by the cantilever from the thermal bath. For the investigation of noise at higher harmonics, *f*_0_, *k*_0_ and *Q*_0_ would have to be replaced by the respective modal values *f_n_*, *k_n_* and *Q_n_* (see Section 2 of Supporting Information File 1). The noise spectral density 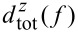 as defined in Equation 1 is displayed in Figure 3a for typical experimental parameters exhibiting a sharp peak at *f*_0_ = 70 kHz (note the logarithmic scale of the ordinate) sticking far out of the white-noise floor 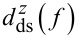 when using low-noise detection electronics. The sharp peak in 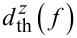 results from the cantilever resonance. Especially high *Q*-factor cantilevers strongly amplify the white spectral power of thermal excitation only in a narrow range of frequencies around *f*_0_ according to Equation 2. The detection-system noise represented by 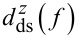 is governed by the quality of the optical and electronic components used in the detection system. In contrast to thermal noise, which is a fixed quantity for a given cantilever and temperature, the detection-system noise floor can be reduced by technical improvements of the detection system [3,7–8].

### Frequency-shift noise

The frequency demodulator of the NC-AFM system extracts the cantilever response to the tip–surface interaction from the sidebands of the cantilever-oscillation frequency spectrum (see Figure 2) and yields the signal power spectral density present in the sidebands, i.e., the displacement power spectral density *D^z^*(*f*) is transformed to the frequency-shift power spectral density *D*^Δ^*^f^*(*f_m_*) by the demodulation process. The noise contribution 
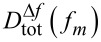
 in this spectrum is the most relevant noise figure in NC-AFM measurements and can be calculated from the demodulator input noise by applying the appropriate demodulator transfer function and an approximation to obtain a simple yet accurate expression for the thermal-displacement power spectral density 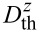. As the frequency noise is represented as a function of the modulation frequency *f_m_*, it is desirable to represent the displacement noise as a function of *f*_0_ ± *f_m_*. For 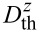, we use the following approximation [1] instead of the precise result from Equation 2:

[3]
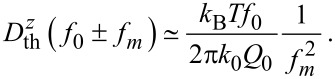


This expression is a very good approximation for modulation frequencies *f_m_* exceeding the cantilever corner frequency *f*_0_/(2*Q*_0_) as seen in Figure 3b. This approximation covers most of the practically relevant spectral range as the corner frequency is smaller than 1 Hz for high-*Q* cantilevers. Combining Equation 1 and Equation 3 yields a simple yet accurate expression for the power spectral density of the total displacement noise in an FM-AFM system operated under high-Q conditions [4]:

[4]
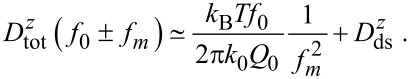


To obtain the noise power spectral density of the frequency-shift signal present at the demodulator output, the demodulator amplitude response for noise 
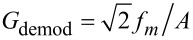
 is applied, and we find [4]

[5]
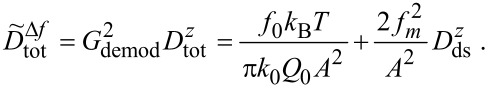


As apparent from Equation 5, the contribution of the thermal noise to the total noise is independent of the modulation frequency *f_m_*, whereas the detection-system-noise power contribution is amplified by the square of the modulation frequency. We further note that the total noise power in Δ*f* depends on the reciprocal of the squared cantilever oscillation amplitude.

**Figure 4:**
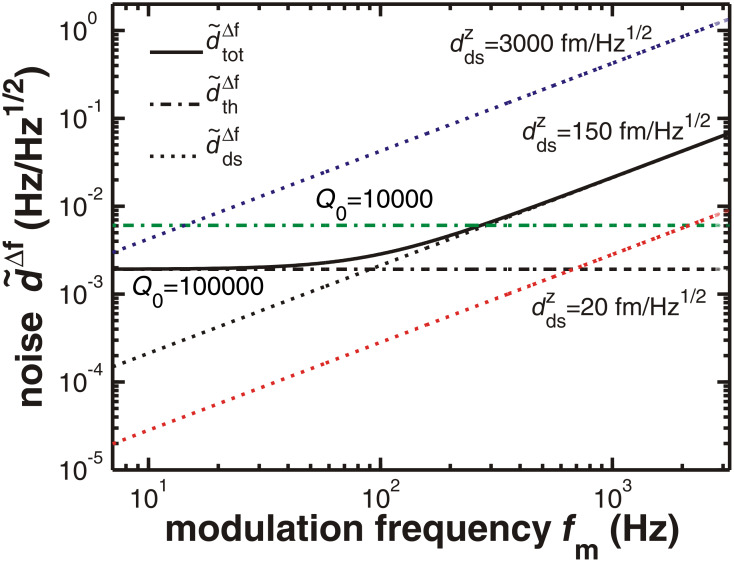
Illustrative representation of the noise spectral density for the total frequency-shift noise 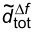 = 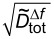 for a system without bandwidth limitations. The total noise is composed of contributions from the thermal noise 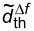 = 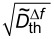 plotted for different *Q*-factors and the noise of the frequency-shift detection system 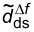 plotted for different values of the noise floor 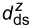 = 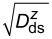. Cantilever and oscillation parameters are *f*_0_ = 70 kHz, *k*_0_ = 2.5 N/m and *A* = 10 nm.

The frequency-shift noise spectral density 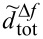 and its components as described in Equation 5 are shown as a function of the modulation frequency *f_m_* in Figure 4 for typical experimental conditions neglecting bandwidth limitations. This result clearly points to the experimental parameters determining the frequency-shift noise: the thermal limit is defined by the temperature *T* and cantilever properties, namely the ratio *f*_0_/(*k*_0_*Q*_0_). For a cantilever with given *f*_0_ and *k*_0_, it is most important to yield a high effective *Q*-factor that may considerably differ from the intrinsic *Q*-factor [9] if one is interested in reducing the thermal-noise limit to the lowest possible value. The noise contribution from the detection system depends on the required bandwidth *B* (range of *f_m_*) and the quality of the detection system represented by 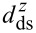. Overall, 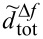 scales with the inverse of the cantilever oscillation amplitude *A*. In Figure 4, the thermal noise limit is shown for typical cantilever properties and for *T* = 300 K as dash-dotted lines representing different *Q*-factors. From Figure 4 we can deduce the displacement noise floor of the detection system 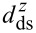 that must not be exceeded for a thermal-noise-limited measurement. We define the bandwidth 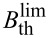 for a thermal-noise-limited measurement by the frequency where the contributions of 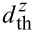 and 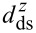 to the total frequency-shift noise spectral density 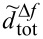 are equal. This frequency 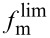 corresponds to the crossing point between the dashed and dash-dotted lines in Figure 4.

It follows that operation at the thermal noise limit can only be obtained if the bandwidth *B* of the demodulator is set close to

[6]
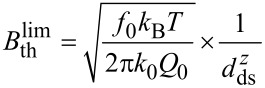


where the noise spectral density 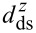 is treated here as a constant. This is fully justified by its white-noise character around the cantilever resonance. Note that this bandwidth limitation is solely based on noise considerations and does not reflect other bandwidth requirements, such as the stable operation of the PLL. However, there is a bandwidth limitation in any real system and 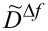 has to be considered as a hypothetical quantity that is rarely accessible. In any PLL system of practical use, the detection bandwidth is defined by internal filters, loop-gain settings and time constants that are normally accessible to the user for an optimisation of the signal processing. Thus, a complete PLL is modelled by using *G*_PLL_ = *G*_filter_ × *G*_demod_, with *G*_filter_ being the amplitude response for the aforementioned filters (see Figure 1). Taking the amplitude response of the full PLL system into account, we obtain for the accessible noise power spectral density

[7]
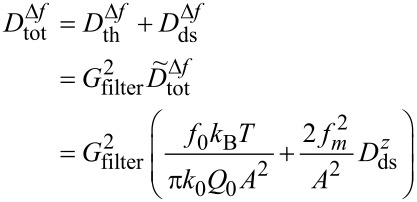


at the output of the bandwidth-limited PLL system. The experimental determination of an unknown amplitude response *G*_filter_ is described in Section 3 of Supporting Information File 1. To characterise the demodulator output noise with a single number, we define δ*f*_tot_ as the root mean square (RMS) of the overall frequency-shift noise:

[8]
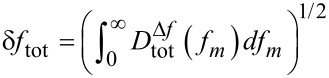


where the integration can practically be limited to an upper frequency limit related to the detection bandwidth *B*. This is fully justified as filtering in the demodulator always yields a low-pass characteristic. A discussion of the RMS noise figure and its calculation by using approximations for the demodulator bandwidth is presented in Section 4 of Supporting Information File 1.

## Experimental

Noise measurements are performed with three NC-AFM systems, named systems A, B and C in the following. All systems are well decoupled from mechanical vibrations by spring suspension and eddy-current damping systems. As an additional precaution, connections between the electronics and piezos are removed during noise measurements to ensure that measurements are not affected by any spurious electrical signals exciting the cantilever. All systems investigated here are based on the optical beam-deflection scheme for measuring the cantilever displacement. Therefore, the laser-light power *P*_pd_ reaching the photodetector is a parameter characterising the system. *P*_pd_ is calculated from the sum signal of the PSD, which in turn depends on the spectral sensitivity of the photodiode, the used laser light wavelength and the DC transimpedance of the preamplifier. The calibration of the detection system is described in Section 1 of Supporting Information File 1.

**System A** is a room-temperature UHV AFM/STM (Omicron NanoTechnology GmbH, Taunusstein, Germany) equipped with an easyPLL (Nanosurf AG, Liestal, Switzerland) for frequency demodulation. The AFM/STM setup has been modified by replacing the light source (light-emitting diode exchanged with a laser diode) and using optimised preamplifiers. Preamplifiers have been optimised for low-noise operation at frequencies around 100 kHz and 300 kHz, respectively, and are exchanged depending on the eigenfrequency of the cantilever. Details on this modification and the frequency response of the preamplifiers can be found in [7]. The light source is a 48TE-SOT (Schäfter+Kirchhoff GmbH, Hamburg, Germany) and emits light at a wavelength of 685 nm, while the PSD has a spectral sensitivity of 0.45 A/W at this wavelength. Noise spectra are recorded with an SR770 spectrum analyser (Stanford Research Systems, Inc., Sunnyvale, CA, USA).

**System B** is a UHV VT AFM/STM (Omicron NanoTechnology GmbH, Taunusstein, Germany) equipped with an easyPLL plus (Nanosurf AG, Liestal, Switzerland) as the demodulator. This system uses a light source having a wavelength of 830 nm, while the spectral sensitivity of the PSD is 0.57 A/W at this wavelength. Noise spectra are measured using the zoom FFT module of a HF2LI lock-in detector (Zurich Instruments AG, Zurich, Switzerland) for spectral analysis.

**Figure 5:**
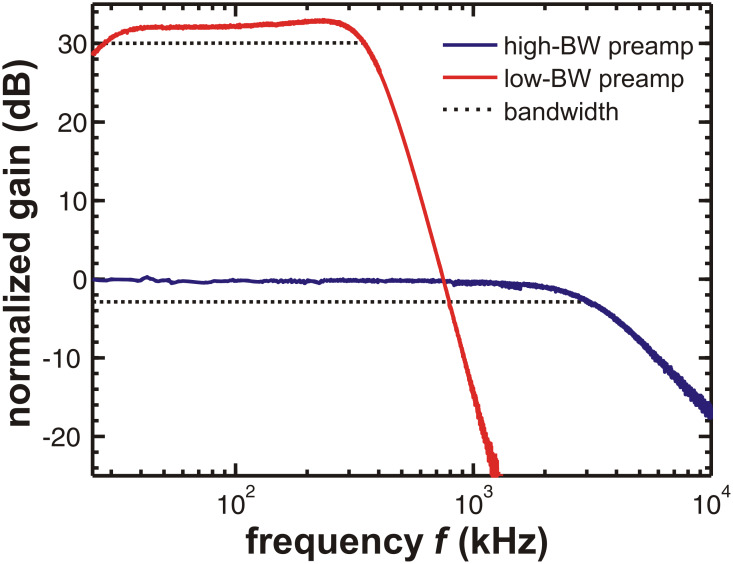
Frequency response of the high-bandwidth preamplifier (bandwidth 3.1 MHz) and the low-bandwidth preamplifier (bandwidth 320 kHz) for System C. The gain is normalised to the DC gain of the high-bandwidth preamplifier. Instead of connecting to the PSD, a sine wave of 0.5 V RMS amplitude was fed into a single quadrant input with a 100 kΩ resistor resulting in 5 μA RMS current.

**System C** is a UHV 750 variable temperature STM/AFM with a PLLPro2 (software version 0.20.0) as the demodulator (RHK Technology, Inc., Troy, MI, USA). The light source is a laser source type 51nanoFCM (Schäfter+Kirchhoff GmbH, Hamburg, Germany) operated in the constant-power mode with radio-frequency modulation to reduce the coherence length to about 300 μm. The laser-light wavelength is 639 nm, and a maximum output power of 5 mW is available at the fibre end while the PSD has a spectral sensitivity of 0.4 A/W at this wavelength. A home-built preamplifier (low-bandwidth preamplifier) or the preamplifier supplied by the manufacturer (high-bandwidth preamplifier) is used depending on the bandwidth requirements. The frequency response of both preamplifiers is shown in Figure 5. To measure noise spectra, the SR770 spectrum analyzer (Stanford Research Systems, Inc., Sunnyvale, CA, USA) is used.

**Force sensors** are commercial silicon cantilevers (Nanoworld AG, Neuchâtel, Switzerland). For our investigations, we use a set of cantilevers covering a large range of resonance frequencies, *Q*-factors and stiffness, to explore the impact of these parameters on the noise figures. Cantilever specifications are compiled in Table 1 and Table 2 (cantilevers D and AO are type FM, cantilevers AQ, AR and V are type NCH, cantilever AP is type NCVH and cantilever AL is type Arrow™ according to the commercial classification scheme).

**Table 1:** Fundamental properties of the cantilevers used for noise analysis. Length *l*, mean width 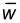 and thickness *t* are provided by the manufacturer. The stiffness *k*_dim_ is calculated from the cantilever dimensions [10]. Typical properties of a qPlus sensor are taken from [11] for comparison.

cantilever	*l* (μm)	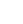 (μm)	*t* (μm)	*k*_dim_ (N/m)

AO 3	224	30	3.0	3.0 ± 0.9
D 5	229	30	2.9	2.5 ± 0.8
AR 17	127	27	3.6	26 ± 5
V 15	125	26	3.7	29 ± 6
AQ 10	123	29	4.5	60 ± 10
AP 5	40	24	2.0	130 ± 50
AL 3	35	42	0.7	9 ± 3^a^
qPlus	2400	126	214	1800

^a^Value provided by the manufacturer.

The cantilever eigenfrequencies of the fundamental and the first and second harmonic mode are determined by measuring resonance curves and fitting the amplitude response function to the data as described in [9]. This procedure also yields quality factors *Q_n_*, while the stiffness *k*_dim_ is calculated from cantilever dimensions and material properties [10] and used as a good approximation to the modal stiffness *k*_0_ [12].

## Results and Discussion

**Figure 6:**
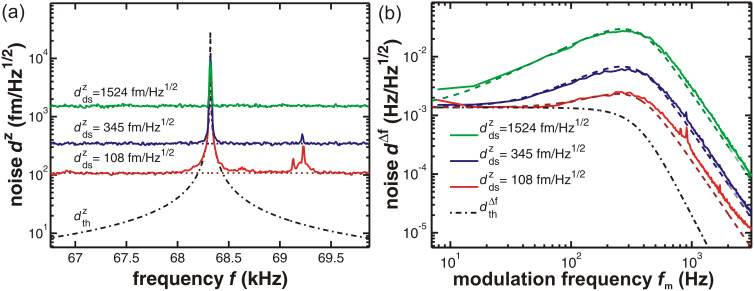
Measured and modelled noise figures for system C. (a) Different levels of displacement noise spectral density 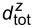 at the output of the low-bandwidth preamplifier for a thermally excited cantilever. Solid lines represent measured data while dotted lines indicate the corresponding detection-system noise floor 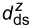. The dash-dotted line is a calculation of the displacement-thermal-noise spectral density 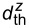 of the cantilever. (b) Calculated noise spectral density 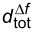 at the PLL output for a cantilever oscillation amplitude of 5 nm and different noise floor levels (dashed lines) compared to measured data (solid lines). The dash-dotted line represents the modelled thermal noise contribution 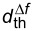 to the noise in the Δ*f* signal. Measurements are performed with cantilever D 5 (see Table 1 and Table 2 for cantilever properties). Filter settings are *f_c_* = 500 Hz, *o* = 3, *P* = −2.0 Hz/deg and *I* = 1 Hz (see Section 3 of Supporting Information File 1 for a detailed explanation).

The noise analysis is performed in two steps. First, we measure the displacement noise spectral density 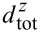 and, second, we investigate how it is propagated to the frequency-shift noise spectral density 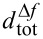. The displacement noise is measured by a spectrum analyser connected directly to the output of the preamplifier (see Figure 1). The measurement range of the spectrum analyser is set to a few kilohertz around the cantilever resonance frequency to obtain high spectral resolution. The spectral density of the noise in the signal *V_z_* is measured and converted to the displacement-noise spectral density 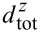 in units of fm/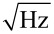 by the calibration procedure outlined in Section 1 of Supporting Information File 1. Figure 6 shows a representative result obtained with system C. The measured displacement noise spectral density is shown in Figure 6a (solid lines), together with the thermal noise contribution 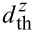 (dash-dotted line) calculated from the given cantilever properties by using Equation 2. The noise floor of the detection system 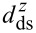 (dotted lines) is measured beside the resonance peak where thermal noise becomes negligible (solid and dashed lines are identical). For a study on how the noise of the detection system 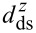 propagates through the demodulation system, different noise levels are artificially created by using white noise from a waveform generator DS345 (Stanford Research Systems, Inc., Sunnyvale, CA, USA) added to the displacement signal *V_z_*. The curve with 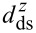 = 108 fm/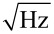 represents the noise floor of the setup while the other curves show artificially increased noise levels.

To measure the frequency-shift noise 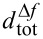, the cantilever is excited to an oscillation with typically 10 nm amplitude and the spectrum analyser is connected to the output of the demodulator (see Figure 1) to measure the voltage noise in the Δ*f* signal. The demodulator is adjusted to zero mean frequency shift and the measurement range of the spectrum analyser is set to the frequency region between 0 and 3 kHz. The measured voltage noise is multiplied by the known conversion factor of the demodulator (e.g., 30 Hz/V) to obtain the frequency-shift-noise spectral density 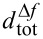 in units Hz/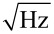. In Figure 6b, this quantity is shown for the same three levels of artificial detection-system displacement noise 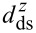 at the input of the demodulator as supplied for the measurement in Figure 6a. Measurements (solid lines) are compared to calculated curves (dashed lines) based on the 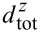 values obtained from the measurements shown in Figure 6a. The curve 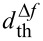 (*f_m_*) is determined from the cantilever properties and the filter settings of the PLL demodulator (thermal contribution in Equation 7) and represents the ideal case of the thermal noise of the cantilever without any detection-system noise. The trailing edge on the right side is caused by the attenuation through the low-pass filter with amplitude response *G*_filter_ (see Section 3 of Supporting Information File 1 for details).

The dashed lines are model curves calculated using Equation 7 with the measured noise contribution 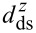 and the calculated thermal noise contribution 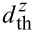. The measured noise curves (solid lines) are in good agreement with the model (dashed lines). A disturbing side peak, which can be observed on top of 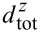 in Figure 6a for a low detection-system noise floor, similarly appears in the corresponding curve 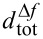 in Figure 6b. Such peaks are due to electromagnetic emission from switching power supplies and other devices present in the laboratory environment. As the propagation of displacement noise 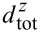 to frequency-shift noise 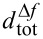 is well reproduced by experimental data, the latter can be used to obtain the level of the noise floor 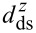 from a noise measurement in the low-frequency region of the Δ*f* signal. By inversion of Equation 7, the displacement-noise spectral density 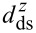 = 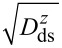 can be obtained from 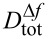 if the system frequency response and 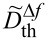 are known:

[9]
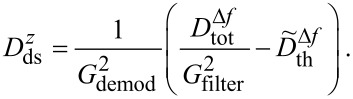


At the frequency *f_m_* = 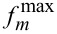, where *f_m_* × *G*_filter_(*f_m_*) has its maximum, the total noise is dominated by the noise from the detection system. At this point, Equation 9 can be simplified when assuming 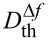 << 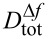 yielding

[10]
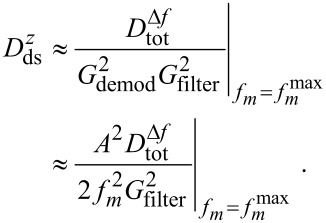


This approximation defines an upper limit 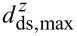 = 
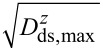
 for the detection-system noise spectral density.

In this manner, we investigate the noise characteristics of the three NC-AFM systems using different preamplifiers and various cantilevers at different eigenmodes; the corresponding results are listed in Table 2. The detection noise measured directly in the displacement signal *V_z_* as shown in Figure 6a is denoted as 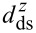 while the same quantity obtained from the frequency-shift noise 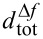 by using Equation 9 is denoted as 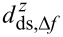. The upper limit derived from Equation 10 is denoted as 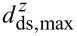. The latter is a useful approximation that can easily be calculated without knowledge of the cantilever properties.

**Table 2:** Cantilever properties and noise figures for systems A, B and C. *f_n_* and *Q_n_* are the eigenfrequencies and *Q*-factors for the *n*th eigenmode of the cantilever. Noise-floor values 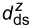 are directly determined from the displacement signal *V_z_*, while 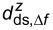 and 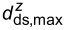 are extracted from the Δ*f* noise at the demodulator output as described in the main text. *P*_pd_ is the total light power on the PSD. For system C, measurements are performed with two different preamplifiers. Missing 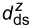 values are due to frequency-range limitations of the spectrum analyser. In the case of higher harmonics, we cannot easily calculate the modal cantilever stiffness, as it strongly depends on the tip mass, which is generally not known [13]. Therefore, determining 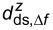 requires the knowledge of the stiffness and is, thus, only calculated for the measurements at the fundamental resonance frequency. Typical properties of a system operated with a qPlus sensor are taken from [11] for comparison.

cantilever	*f_n_*	*Q_n_*	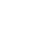	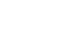	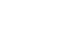	*P*_pd_
			(fm/ 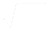 )	(fm/ 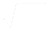 )	(fm/ 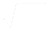 )	(μW)

System A						

AQ 10	*f*_0_ = 361,599 Hz	*Q*_0_ = 21,200		275	278	97

System B						

V 15	*f*_0_ = 279,451 Hz	*Q*_0_ = 47,200	125	119	124	105

System C, low-bandwidth preamplifier						

D 5	*f*_0_ = 68,353 Hz	*Q*_0_ = 118,000	115	122	130	120
AO 3	*f*_0_ = 68,183 Hz	*Q*_0_ = 173,700	237	223	226	106
AR 17	*f*_0_ = 276,360 Hz	*Q*_0_ = 39,200		97	98	120

System C, high-bandwidth preamplifier						

AO 3	*f*_0_ = 68,183 Hz	*Q*_0_ = 173,700		416	417	105
AO 3	*f*_1_ = 437,086 Hz	*Q*_1_ = 48,500			93	105
AO 3	*f*_2_ = 1,235,138 Hz	*Q*_2_ = 15,200			51	105
AR 17	*f*_0_ = 276,360 Hz	*Q*_0_ = 39,200		258	259	120
AR 17	*f*_1_ = 1,730,811 Hz	*Q*_1_ = 6,300			99	120
AP 5	*f*_0_ = 1,996,199 Hz	*Q*_0_ = 32,400		302	309	77
AL 3	*f*_0_ = 1,316,757 Hz	*Q*_0_ = 16,600		845	892	18

qPlus system						

qPlus	*f*_0_ = 32,768 Hz	*Q*_0_ = 5,000	62			

Table 2 allows a comparison of the noise floor for different NC-AFM systems and demonstrates the influence of cantilever properties on the noise figures. The best values for the noise floor achieved here are around 100 fm/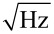 as measured for cantilever V 15 in system B and cantilevers D 5 and AR 17 in system C. These cantilevers have a length in the range of 100 μm to 250 μm. Exchanging the preamplifier may cause a large difference in the noise floor. This can be observed for cantilevers AO 3 and AR 17 in system C where the noise floor is doubled by changing from the low-bandwidth to the high-bandwidth preamplifier. The benefit of the high-bandwidth amplifier is the possibility to operate cantilevers at their higher resonance frequencies, where the displacement-noise floor significantly decreases even for a similar voltage noise caused by the laser-power-dependent photodiode shot noise due to different amplitude calibration factors for the corresponding modes. However, due to the length of only 40 μm and 35 μm, for the high-frequency cantilevers AP 5 and AL 3, the laser adjustment becomes difficult, yielding only 77 μW and 18 μW laser power on the PSD compared to about 100 μW for other cantilevers. Therefore, the detection-noise floor for these cantilevers is much higher than for larger cantilevers. With an improved laser-spot adjustment, however, a noise floor close to 100 fm/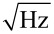 should be possible.

**Figure 7:**
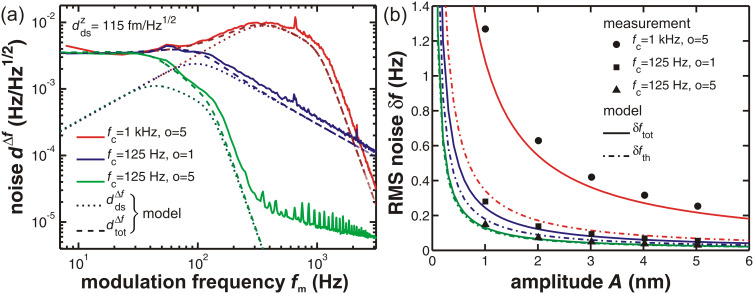
(a) Measured (solid) and modelled (dashed) frequency-shift-noise spectral density 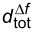 using three different filter settings. Dotted lines show the contribution 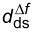 of the detection-system noise to the total frequency-shift-noise spectral density for a noise floor of 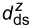 = 115 fm/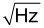 at the input of the demodulator. The oscillation amplitude is 5 nm. (b) Noise figures for different cantilever oscillation amplitudes. Measured RMS frequency-shift noise δ*f* (circles, squares, triangles) for different PLL filter settings compared to predictions from Equation 8 (solid lines) using the measured detection-system noise 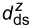 in the cantilever displacement signal *V_z_*. Dash-dotted lines represent calculations of the thermal-noise contribution δ*f*_th_. Measurements are performed with cantilever D 5 in system C (see Table 1 and Table 2 for cantilever properties).

In Figure 7, we illustrate the choice of optimum filter settings for a thermal-noise-limited detection. For that purpose, the frequency noise originating from the same detection system but passed through different PLL filters is shown. As these measurements are performed in system C, the filters are modelled as a closed loop where the settings of the PI controller have a significant effect on the frequency response and need to be individually adjusted for each setting of the loop filter order *o* and cutoff frequency *f_c_* (see Section 3 of Supporting Information File 1 for details). The optimum settings for each loop filter used in the following are listed in Table S4 in Section 3 of Supporting Information File 1. In Figure 7a, we display the noise spectral characteristics of the Δ*f* signal, while Figure 7b shows a plot of the total noise represented by the RMS value of the Δ*f* signal as a function of the cantilever oscillation amplitude. Using a bandwidth of *B*_−3dB_ = 385 Hz (*f_c_* = 1 kHz, *o* = 5), the total noise exceeds the thermal noise level by half an order of magnitude. Choosing a much lower bandwidth of *B*_−3dB_ = 48 Hz (*f_c_* = 125 Hz, *o* = 5) decreases the frequency range where the signal is not attenuated below the one defined by the thermal noise limit. The optimum filter setting for the 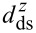 noise floor present in this measurement is a filter setting with a bandwidth of *B*_−3dB_ = 103 Hz (*f_c_* = 125 Hz, *o* = 1), where the total noise does not significantly exceed the thermal noise and the signal is not unnecessarily attenuated. For all filter settings investigated here, experiment (solid lines) and model (dashed lines) agree well with each other.

In Figure 7b, measured values δ*f*_tot_ (circles, squares, triangles) are compared to calculated values δ*f*_tot_ derived from Equation 8 (solid lines) and δ*f*_th_ defining the thermal limit of the RMS frequency-shift noise (dash-dotted lines). While there is a large difference between thermal noise and total noise for the large-bandwidth filter setting (*f_c_* = 1 kHz, *o* = 5), this discrepancy becomes smaller and finally negligible on further reduction of the bandwidth. Note, however, that the settings yielding the smallest RMS noise are not the optimum as the corresponding filter does not only reduce the noise but attenuates the NC-AFM signal more than necessary for thermal-noise-limited operation.

The RMS value of the total noise is an important figure of merit of the NC-AFM detection system, as it defines the minimum detectable frequency shift. Figure 7b is an excellent demonstration of the potential of small amplitudes for atomic resolution measurements as it is known that the atomic contrast increases with reduced cantilever oscillation amplitude [11,14]. For a measurement with *B*_−3dB_ = 385 Hz (red line, *f_c_* = 1 kHz, *o* = 5), one would choose an amplitude of 5 nm or above to reduce the noise; however, this would also reduce the atomic contrast compared to a lower amplitude measurement. For a measurement with *B*_−3dB_ = 103 Hz (blue line, *f_c_* = 125 Hz, *o* = 1), one can take full advantage of the increased atomic corrugation for the smaller amplitude, as the total noise is even below the thermal noise for the larger amplitude.

**Figure 8:**
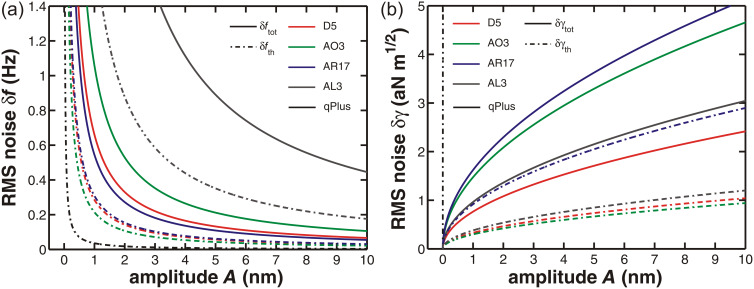
(a) RMS frequency-shift noise δ*f* and (b) normalised RMS frequency-shift noise δ*γ* in the limit of purely thermal noise (dash-dotted lines) as well as in combination with the corresponding detection noise (solid lines) for different cantilevers (*T* = 300 K, filter settings *f_c_* = 500 Hz, *o* = 3, *P* = −2.0 Hz/deg and *I* = 1 Hz yielding *B*_−3dB_ = 258 Hz). Calculations are performed for the fundamental eigenfrequency *f*_0_ of the cantilever. Cantilever properties and the corresponding values of the displacement-noise floor of the detection system 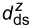 are given in Table 1 and Table 2.

In Figure 8a, different cantilevers are compared regarding their total RMS frequency shift noise δ*f*_tot_ (solid lines) as well as the thermal frequency noise δ*f*_th_ (dash-dotted lines). Here, the same bandwidth of *B*_−3dB_ = 258 Hz is chosen for all simulations to facilitate the comparison of the cantilevers. Regarding thermal noise, all cantilevers except AL 3 exhibit an RMS noise below 0.5 Hz for amplitudes larger than 1 nm. The total noise values are ordered by the level of the corresponding noise floor 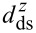, dominating the total noise for a bandwidth larger than the thermal-limit bandwidth. Note that the thermal-noise contribution of AL 3 is even larger than the total noise of the other cantilevers. These results are compared to typical values for a qPlus sensor with parameters taken from [11]. The thermal noise δ*f*_th_ of the qPlus sensor is an order of magnitude below the values for the cantilevers. Including the noise of the detection system, δ*f*_tot_ of the qPlus sensor is nearly identical to the thermal noise δ*f*_th_ obtained for cantilever D 5 (curve not shown) and, therefore, only half of the noise level of the best cantilevers.

For a valid comparison of measurements obtained under different experimental conditions, however, it is important to compare limits in the normalised frequency shift *γ* rather than the plain frequency shift Δ*f*. Based on the concept of the normalised frequency shift [15], we define a normalised-frequency-shift RMS noise as

[11]
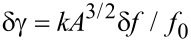


to compare the noise characteristics of cantilevers independently of their stiffness and resonance frequency and display the corresponding data as a function of the cantilever oscillation amplitude in Figure 8b. Regarding the thermal contribution δγ_th_ to the normalised frequency-shift noise, cantilevers D 5 and AO 3 exhibit the best performance but are closely followed by cantilever AL 3. The δγ_th_ value of AR 17 is even larger than the total noise δγ_tot_ of cantilever D 5. This is presumably due to the large ratio *k*/*f*_0_. Although cantilever AL 3 has the largest detection-system noise floor, its δγ_tot_ is quite close to that of cantilever D 5. On the other hand, the qPlus sensor has a noise level δγ more than two orders above the results for the cantilevers due to its exceptional *k*/*f*_0_ ratio. Therefore, the advantageous noise figures of the qPlus sensor documented in Figure 8a can only be exploited if the sensor is operated at very low amplitudes.

As cantilevers D 5 and AL 3 have thermal bandwidth limits of 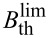 = 95 Hz and 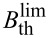 = 85 Hz according to Equation 6, they are best suited for thermal-noise-limited operation. Operating them with a filter *B*_−3dB_ = 103 Hz (*f_c_* = 125 Hz, *o* = 1) yields noise limits of δγ_tot_ = 0.69 aN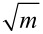 and δγ_tot_ = 0.84 aN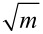, respectively, for an oscillation amplitude of *A* = 5 nm. Assuming, the detection noise floor of AL 3 could be decreased to 130 fm/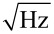 as for cantilever D 5, thermal-noise-limited operation with a bandwidth of 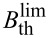 = 586 Hz and δγ_tot_ = 1.62 aN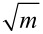 would be possible for an oscillation amplitude of 5 nm and a filter setting of *B*_−3dB_ = 646 Hz (*f_c_* = 1000 Hz, *o* = 3). This means that by switching from cantilever D 5 to AL 3, the usable bandwidth could be increased by a factor of six at the cost of increasing δγ_tot_ by a factor of two. In comparing such numbers, one should, however, consider that the assumed oscillation amplitude of 5 nm may be at the limit of stable operation [15], specifically for the soft cantilever D 5. In conclusion, the high-frequency and relatively stiff cantilever AL 3 represents an excellent choice for high-speed measurements with small amplitudes and good noise performance, while the larger and softer cantilever D 5 is the better choice for slower measurements with best possible noise performance.

## Conclusion

We investigated the relation between the displacement noise in NC-AFM measurements and the corresponding frequency-shift noise at the output of the demodulator and demonstrated that predictions based on the demodulator transfer function and filtering are well reproduced by experiments. For a quantitative analysis of the noise, a precise amplitude calibration of the detection system relating electrical signals to the mechanical oscillation of the cantilever is inevitable. The displacement noise of an NC-AFM system can be measured directly with a spectrum analyser at the output of the detection system, and the thermal component of the displacement noise extracted from such spectra agrees well with spectra derived from a model of thermal cantilever excitation. The noise contribution of the detection system can be obtained from the white-noise floor of the measured spectra. The knowledge of the detection-system transfer functions allows one to predict the frequency-shift noise from the measured displacement noise, and by inversion, a measurement of the detection-system noise from the frequency-shift noise is possible. While the former analysis requires a spectrum analyser with very high resolution and an operating range that includes the eigenfrequency of the cantilever, the latter procedure requires only a measurement of the frequency-shift noise with a device covering the frequency range between a few hertz and about 10 kHz at moderate frequency resolution. Therefore, a rather complete noise characterisation with a simple spectrum analyser as integrated in many NC-AFM systems is possible for a calibrated system.

The framework of modelling noise in the NC-AFM system in combination with the experimental practice described here provides a clear guideline for system design and the choice of experimental parameters for thermal-noise-limited operation. The analysis shows that for a noise-optimised NC-AFM measurement, the right choice of the cantilever is most important, and obtaining a high effective *Q*-factor should be given great attention to keep the level of thermal noise at a minimum. The bandwidth of thermal-noise-limited operation is determined by the noise generated in the detection system. By an appropriate choice of PLL filter settings, one can make full use of this bandwidth without attenuating the NC-AFM signal while very efficiently eliminating most of the detection system noise. We find that with a technically optimised system and an appropriate choice of experimental parameters, room-temperature thermal-noise-limited NC-AFM measurements are possible over a bandwidth of 100 Hz and a detection limit smaller than 0.7 aN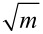 for the normalised frequency shift operating at an amplitude of 5 nm.

## Supporting Information

File 1 Experimental details and theory.
