# Supplementary material for: Thermal noise limit for ultra-high vacuum noncontact atomic force microscopy
Source: Beilstein J Nanotechnol. 2013 Jan 17;4:32–44. doi: 10.3762/bjnano.4.4 (PMC3566860; doi:10.3762/bjnano.4.4)
Supplement: File 1 — Experimental details and theory. [file Beilstein_J_Nanotechnol-04-32-s001.pdf]

Supporting Information

for

Thermal-noise limit for ultra-high vacuum noncontact atomic force microscopy

Jannis Lübbe[1], Matthias Temmen[1], Sebastian Rode[2,3], Philipp Rahe[2,4], Angelika Kühnle[2] and Michael Reichling*[1]

Address: [1]Fachbereich Physik, Universität Osnabrück, Barbarastraße 7, 49076 Osnabrück, Germany; [2]Institut für Physikalische Chemie, Johannes Gutenberg-Universität Mainz, Duesbergweg 10-14, 55099 Mainz, Germany; [3]now at: SmarAct GmbH, Schütte-Lanz-Strasse 9, 26135 Oldenburg, Germany and [4]now at: Department of Physics and Astronomy, The University of Utah, 115 South 1400 East, Salt Lake City, UT 84112, USA

Email: Michael Reichling - reichling@uos.de

* Corresponding author

Experimental details and theory

1 Amplitude calibration

In the optical beam-deflection detection scheme, the cantilever oscillation yields a nearly harmonically oscillating voltage V_z at the output of the photodetector preamplifier as illustrated in Figure S1. For the calibration, we relate the voltage amplitude V_A of the oscillating voltage $V_z = V_A \sin(2\pi f t + \phi)$ to the amplitude A of the mechanical cantilever oscillation with frequency f using a procedure where the tip–surface interaction is kept constant at a certain value while A is varied [1]. As the cantilever is inclined at an angle θ towards the sample surface, we distinguish between A, the oscillation amplitude of the cantilever and the component A_z of the oscillation amplitude perpendicular to the sample surface. These two quantities are related by

$$A = A_z \cos\theta \quad . \tag{12}$$

A deflection of the cantilever by an angle $\Delta\theta$ changes the angle of the reflected laser beam by $2\Delta\theta$, resulting in a displacement of the laser spot on the PSD. The displacement on the PSD results in a difference ΔI_z of the photocurrent from the PSD quadrants, which is in turn converted to a voltage signal V_z by the preamplifier. The calibration establishes the relation between the amplitude A of the oscillating cantilever and the voltage amplitude V_A

$$A = S \times V_A \tag{13}$$

where S is the sensitivity or calibration factor. Here, we obtain S in a noncontact measurement without any knowledge of the details of the detection system except the tilt angle θ. To accomplish this, the normalised frequency shift derived from the measured frequency shift Δf [2]

$$\gamma(z_{\mathrm{ts}}, A) = \frac{k_0 A^{3/2}}{f_0} \Delta f(z_{\mathrm{ts}}, f_0, k_0, A) \tag{14}$$

is kept constant during the entire calibration procedure to maintain a certain level of tip–surface interaction independent of the cantilever oscillation amplitude (see main manuscript text and Figure S1

for a description of the quantities involved). Note that this concept is valid only for amplitudes larger than a critical value, which is typically 1 nm [3].

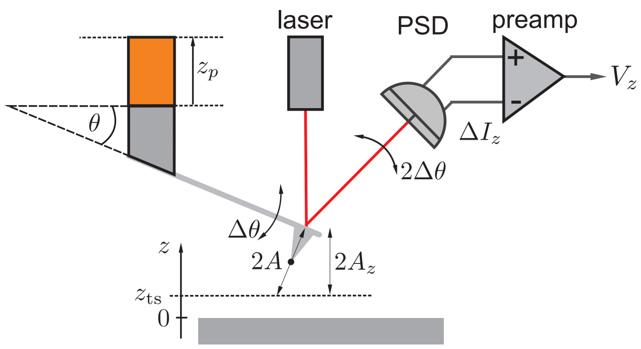

Figure S1: Schematic representation of the signal path in an NC-AFM system based on the optical beam-deflection scheme. The relation between the amplitude A of the oscillating cantilever and its component A_z perpendicular to the surface is illustrated as well as the periodic cantilever deflection $\Delta\theta$, the PSD output current difference ΔI_z and the preamplifier output voltage $V_z = V_A \sin(2\pi f t + \phi)$. The quantity z_{ts} represents the tip–sample distance in the lower turning point of the cantilever oscillation.

In the calibration experiment, A is stepwise increased or decreased by a variation of the amplitude feedback setpoint defining V_A, while the normalised frequency shift γ is kept constant by the choice of an appropriate frequency-shift setpoint Δf_{set}. This causes the topography feedback to readjust the z-piezo position z_p, which is recorded as a function of the varied oscillation amplitude V_A as shown in Figure S1. A MATLAB script (The MathWorks, Inc., Natick, MA, USA) is used to control the setpoints of V_A and Δf and to determine the corresponding z_p value. Initially, the tip is approached to the surface and stabilised at a distance in the long-range interaction regime by the choice of an appropriate frequency shift setpoint Δf_{set}. After waiting for a while to reduce piezo creep effects, the piezo position z_p is recorded for each oscillation voltage amplitude V_A, while $V_A^{3/2}\Delta f$ is kept constant by adjusting the Δf setpoint accordingly. Typical parameters and results are shown in Table S1 and Figure S2, respectively.

Table S1: Typical chart for amplitude calibration compiling voltage oscillation amplitudes V_A and their corresponding frequency shift setpoints Δf_{set} for keeping the normalised frequency shift at a constant value. The resulting response of the topography feedback for a single series of measurements is given as z_p^{up}.

V_A (mV)	Δf_{set} (Hz)	z_p^{up} (nm)
500	−30.00	24.04
511	−29.03	24.21
522	−28.11	24.39
533	−27.23	24.55
554	−26.40	24.73
556	−25.61	24.89
567	−24.86	24.07
578	−24.15	24.23
589	−23.47	24.36
600	−22.82	24.53

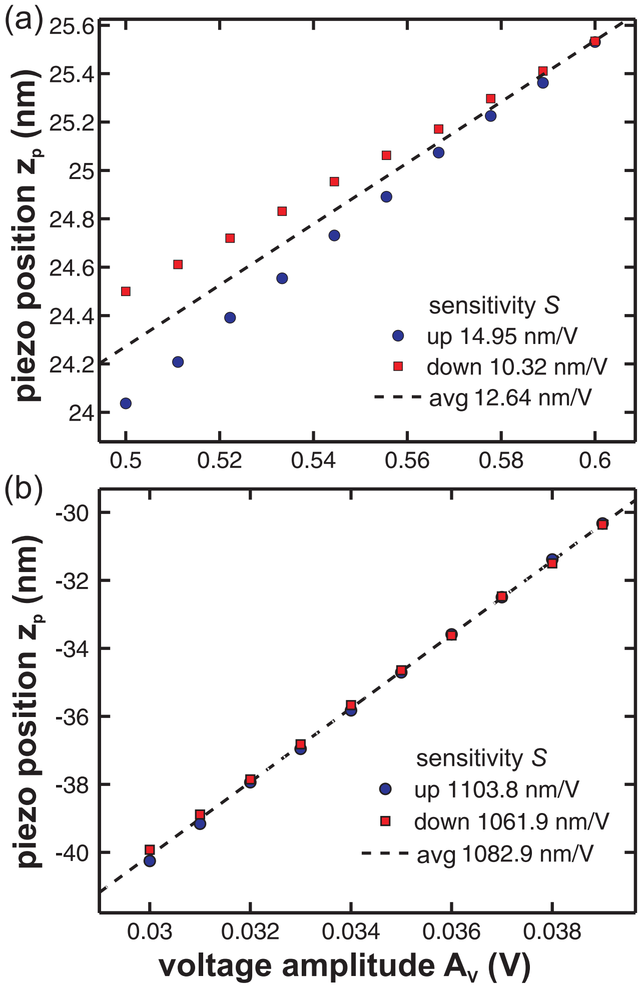

Figure S2: Typical results for a cantilever-oscillation-amplitude calibration performed in system C. (a) Amplitude calibration curves strongly influenced by thermal drift in the z-direction (180 pm/min) yielding different results for runs with increasing and decreasing amplitude. The measurement is taken within 150 s for a cantilever of NCH type ($f_0 = 311420$, $k_{\mathrm{dim}} = 50$ N/m, $Q_0 = 34900$). (b) Calibration curves exhibiting negligible thermal drift. The measurement is taken within 320 s for a cantilever of FM type ($f_0 = 68183$, $k_{\mathrm{dim}} = 3$ N/m, $Q_0 = 173700$). The strongly different sensitivities obtained for measurements (a) and (b) are not only the results of different cantilever properties but mainly stem from the use of preamplifiers with a strongly different gain.

The calibration factor S is obtained from the slope in the plot z_p versus V_A and considering the correction for the tilt angle θ:

$$S = \frac{\Delta z_p}{\Delta V_A} \cos\theta \quad . \tag{15}$$

To compensate for thermal drift as well as piezo creep, this procedure is first performed with increasing oscillation amplitude and then repeated with decreasing oscillation amplitude. Respective measurements are denoted as "up" and "down" in the plots of Figure S2. While Figure S2a shows a measurement with a significant drift in the z direction, for the small drift measurement in Figure S2b, the "up" and "down" curves almost coincide with each other. To reduce the systematic error due to drift, the mean slope is used as the final result. The calibration measurement is repeated several times and results are averaged to reduce the statistical error. The standard deviation for a series of calibration runs is typically 1%.

2 Thermally excited cantilever oscillation

Thermally excited fluctuations of the cantilever are of special interest as they determine the ultimate noise limit in NC-AFM measurements. Based on an approach originally proposed to describe thermal noise in a resistor [4], we derive the power spectral density for the displacement D^z_{th} of a cantilever in thermal equilibrium with a thermal bath at temperature T. According to the equipartition theorem [5], we assume that the mean potential energy of the one-dimensional cantilever oscillator in contact with the thermal bath is equal to $k_{\mathrm{B}}T/2$

$$\frac{1}{2}k\left\langle z^2(t)\right\rangle = \frac{1}{2}k_{\mathrm{B}}T \tag{16}$$

with k being the static stiffness of the cantilever and k_{B} the Boltzmann constant. The cantilever is assumed to be excited by random thermal fluctuations in the thermal bath. According to the Wiener–Khinchin theorem [5], the cantilever mean-square displacement of this random stationary process

can be related to the power spectral density of the cantilever displacement D^z_{th} by

$$\langle z^2(t) \rangle = \frac{1}{2\pi} \int_0^\infty D^z_{\mathrm{th}}(\omega) d\omega \quad . \tag{17}$$

The total mean-square displacement can be decomposed into the sum of displacements originating from the cantilever eigenmodes [6] and is related to constants of modal stiffness k_n for the nth eigenmode. Using Equation 16 we find:

$$\begin{aligned} \frac{1}{2} k \langle z^2(t) \rangle &= \frac{k}{2} \sum_{n=0}^{\infty} \langle z_n^2(t) \rangle \\ &= \frac{k}{2} \sum_{n=0}^{\infty} \frac{k_\mathrm{B} T}{k_n} \\ &= \frac{k_\mathrm{B} T}{2} \sum_{n=0}^{\infty} \frac{k}{k_n} \end{aligned} \tag{18}$$

where z_n is the displacement corresponding to the nth eigenmode. Equation 18 demonstrates that the contribution of the nth eigenmode to the total energy of the thermally excited cantilever is $(k/k_n)(k_\mathrm{B} T/2)$.

It has been shown that the modal stiffness k_n, specifically for the higher harmonics, strongly depends on the mass ratio $\mu = m_{\mathrm{tip}}/m_{\mathrm{beam}}$ [7,8], where m_{tip} and m_{beam} are the masses of tip and cantilever beam, respectively. Calculated relations between the modal stiffness and the static stiffness depending on the mass ratio are given in Table S2 for the first four eigenmodes. The Table shows that 97% of the total oscillation energy is in the fundamental mode for a beam without a tip and this fraction increases as the tip mass increases.

The ratios between the higher harmonic eigenfrequencies f_n to the fundamental eigenfrequency f_0 in dependence of the mass ratio μ are given in Table S3.

Similar to the mean-square cantilever displacement, the total thermal displacement power spectral density is represented as the sum of contributions from the cantilever eigenmodes:

$$D^z_{\mathrm{th}}(\omega) = \sum_{n=0}^{\infty} D^z_{\mathrm{th},n}(\omega) \quad . \tag{19}$$

Table S2: Ratios of modal cantilever stiffness k_n to the static stiffness k calculated for several eigenmodes of a rectangular cantilever beam with a tip for different mass ratios using Equation 5 in [8].

stiffness ratio	mass ratio μ 0	0.01	0.05	0.1
k_0/k	1.03	1.03	1.02	1.01
k_1/k	40.46	43.21	55.83	74.93
k_2/k	317.2	360.0	578.4	949.66
k_3/k	1218	1471	2890	5534

Table S3: Ratios of the eigenfrequency f_n of higher harmonics of a rectangular cantilever beam with a tip to the eigenfrequency f_0 of its fundamental mode for different mass ratios. Values are calculated by using Equation 10 in [9].

frequency ratio	mass ratio μ 0	0.01	0.05	0.1
f_1/f_0	6.27	6.27	6.35	6.52
f_2/f_0	17.55	17.57	17.94	18.71
f_3/f_0	34.39	34.45	35.43	37.30

To determine the displacement power spectral density for each mode n, we assume a constant energy spectral density Ψ_n from the thermal bath exciting the cantilever following the arguments given by Nyquist for the explanation of a thermally excited electrical current in a resistor [4].

The response of the cantilever to this thermal excitation is determined by its amplitude response $G(\omega)$, which is the amplitude response of a damped harmonic oscillator [10]. For each eigenmode we assume

$$G_n(\omega) = \frac{1}{\sqrt{(1-\omega^2/\omega_n^2)^2 + \omega^2/(\omega_n^2 Q_n^2)}} \tag{20}$$

where $\omega_n = 2\pi f_n$ and Q_n are the angular eigenfrequencies and quality factors of the nth mode, respectively. Therefore, we represent the energy spectral density of the nth mode, being $(1/2)kD^z_{\mathrm{th},n}$, as

$$\frac{1}{2}kD^z_{\mathrm{th},n}(\omega) = G_n^2(\omega)\Psi_n \quad . \tag{21}$$

Considering the result of Equation 18, stating that a fraction k/k_n of the total energy $k_\mathrm{B}T/2$ goes to

the nth eigenmode, and integrating over $D^{z}_{\mathrm{th},n}$ we find for each mode:

$$\frac{k}{k_n}\frac{k_{\mathrm{B}}T}{2} = \frac{1}{4\pi}k\int_0^\infty D^{z}_{\mathrm{th},n}(\omega)d\omega$$
$$= \frac{1}{2\pi}\int_0^\infty G_n^2(\omega)\Psi_n d\omega$$
$$= \frac{\Psi_n}{2\pi}\int_0^\infty \frac{1}{(1-\omega^2/\omega_n^2)^2+\omega^2/(\omega_n^2 Q_n^2)}d\omega \quad .$$

We substitute $\zeta_n = \omega/\omega_n$ and $d\omega = \omega_n d\zeta_n$ and find

$$\frac{k}{k_n}\frac{k_{\mathrm{B}}T}{2} = \frac{\Psi_n\omega_n}{2\pi}\int_0^\infty \frac{d\zeta_n}{\zeta_n^4+\left(\frac{1}{Q_n^2}-2\right)\zeta_n^2+1} \quad .$$

Solving the integral using

$$\int_0^\infty \frac{d\zeta_n}{\zeta_n^4+a\zeta_n^2+b} = \frac{\pi}{2}\frac{1}{\sqrt{b}\sqrt{a+2\sqrt{b}}}$$

with $a = (1/Q_n^2-2)$ and $b = 1$ yields the excitation energy spectral density for the nth mode:

$$\Psi_n = \frac{2k_BT}{Q_n\omega_n}\frac{k}{k_n} \quad . \tag{22}$$

Inserting this result in Equation 21 yields the displacement power spectral density of the thermally excited cantilever:

$$D^{z}_{\mathrm{th},n}(\omega) = \frac{4k_{\mathrm{B}}T/(k_nQ_n\omega_n)}{(1-(\omega/\omega_n)^2)^2+\omega^2/(\omega_nQ_n)^2}$$

or

$$D^{z}_{\mathrm{th},n}(f) = \frac{2k_{\mathrm{B}}T/(\pi k_nQ_nf_n)}{(1-(f/f_n)^2)^2+f^2/(f_nQ_n)^2}$$

for the nth oscillation mode. This agrees well with results reported by other authors [11-13]. A

calculated example of the displacement thermal noise spectral density $d^z_{\text{th}} = \sqrt{D^z_{\text{th}}}$ for a typical cantilever at room temperature for the first four oscillation modes is given in Figure S3.

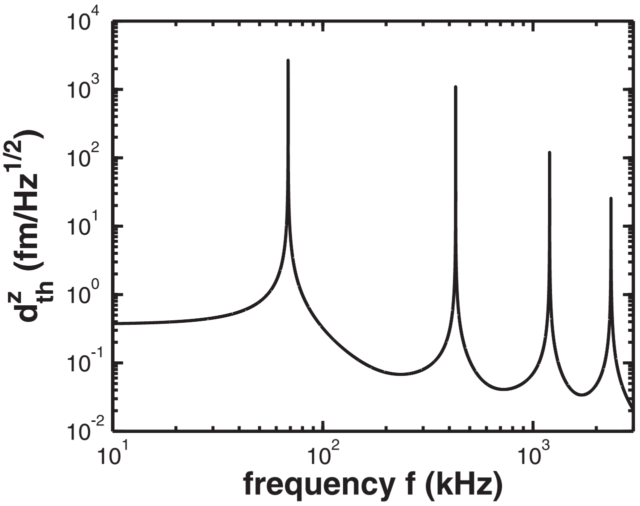

Figure S3: Calculated thermal noise spectral density d^z_{th} in the frequency range covering the first four modes of a cantilever with $f_0 = 68347$ Hz, $k_0 = 2.8$ N/m and $Q_0 = 103600$. Values for f_n and k_n of the higher modes are calculated neglecting the tip mass. The Q_n are typical values experimentally determined for a cantilever of this type ($Q_1 = 39000$, $Q_2 = 5800$, $Q_3 = 2000$).

3 Measuring and controlling the frequency response of the PLL

The effective demodulation bandwidth of the PLL system depends on details of the PLL feedback loop filter settings and output filter characteristics. Hence, the experimental determination of the frequency response is highly desirable unless this information is fully available in the technical documentation of the demodulator system. For a measurement of the amplitude response G_{filter}, the tip is positioned above the sample in range of solely electrostatic forces. The frequency shift Δf is recorded as a function of the sample bias voltage V_{dc} yielding a parabola like the one shown in Figure S4a. Then, the bias voltage is modulated with a constant amplitude of typically 1 V and V_{dc} is fixed at a working point V^0_{dc}, where the bias modulation yields a well measurable modulation in the frequency shift signal. The amplitude Δf^m of the latter signal is determined by a lock-in detector integrated in the NC-AFM setup as shown in Figure S4b. The amplitude response is measured by recording the signal Δf^m while sweeping f_m over the desired frequency range.

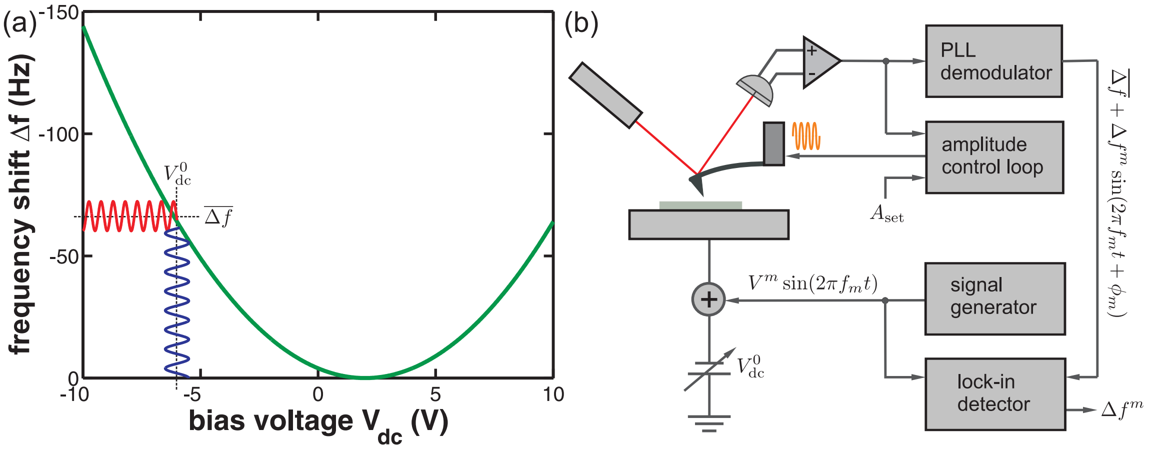

Figure S4: (a) Illustrative representation for a Δf vs V_{dc} curve and (b) experimental scheme for the measurement of the frequency response of the PLL demodulator of an NC-AFM system. The modulation $V^m \sin(2\pi f_m t)$ of the bias voltage V_{dc} yields a modulation of the Δf signal. The bias voltage working point V^0_{dc} is chosen to obtain a frequency shift amplitude of typically $\Delta f^m = 3$ Hz. A signal generator is used to modulate the bias voltage with a constant amplitude V^m while recording the resulting modulation amplitude of the frequency shift signal Δf as a function of the modulation frequency f_m with a lock-in detector synchronised to the signal generator.

Measurements of the frequency response are performed with systems A, B and C, where the demodulation is accomplished by an easyPLL system, an easyPLL plus system, or a PLLpro2 system, respectively, as described in the experimental section of the main text.

In the case of the **easyPLL systems**, second order low-pass filters located at the Δf output of the demodulator with the transfer function

$$H(s) = 1/\left(1 + s/(\omega_c Q_f) + (s/\omega_c)^2\right) \tag{23}$$

are installed, where f_c and Q_f are the cutoff frequency and the quality factor of the filter, respectively. The variable s is the complex angular frequency. The amplitude response is calculated as the absolute value of the complex transfer function for $s = i\omega$

$$G(\omega) = |H(i\omega)|$$

and plotted against the modulation frequency f_m yielding a response curve like the one shown in Figure S5. We also perform a fit of a model curve based on Equation 23 to the experimental data.

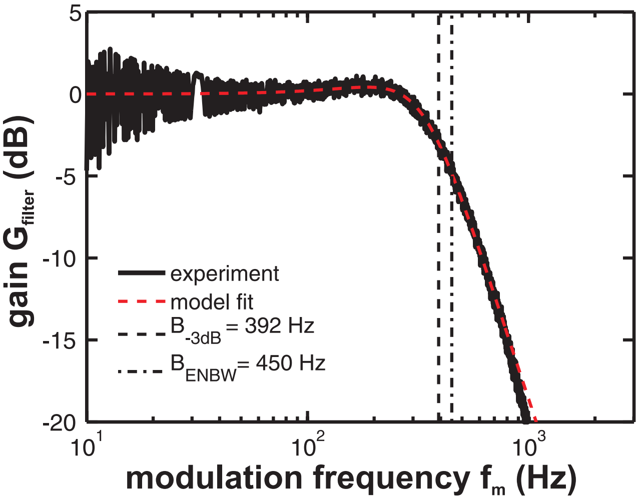

Figure S5: Measured amplitude response of the 300 Hz filter of the easyPLL system modelled as a second order low pass filter. A fit of the model to the experimental data yields $f_c = 338$ Hz and $Q_f = 0.85$.

For the transfer function of the filter having a nominal bandwidth of 300 Hz, the fit yields $f_c =$ 338 Hz and $Q_f = 0.85$ as cutoff frequency and quality factor, respectively.

The bandwidth $B_{-3\mathrm{dB}}$ is defined as the frequency f_m at which the signal is attenuated by 3 dB:

$$G_{\mathrm{filter}}(B_{-3\mathrm{dB}}) = 1/\sqrt{2} \quad .$$

For the 300 Hz filter, we obtain $B_{-3\mathrm{dB}} = 392$ Hz. For noise considerations, often the equivalent noise bandwidth B_{ENBW} defined as [14]

$$B_{\mathrm{ENBW}} = \int_0^\infty G^2_{\mathrm{filter}} d f_m \tag{24}$$

is used instead. Here, we yield $B_{\mathrm{ENBW}} = 450$ Hz for the 300 Hz filter.

For the **easyPLL plus system**, we investigate two different filter settings with nominal frequencies of 120 Hz and 400 Hz and obtain the amplitude response curves shown in Figure <S6 with $f_{c,120} = 120$ Hz, $f_{c,400} = 362$ Hz, $Q_{f,120} = 0.73$ and $Q_{f,400} = 0.83$ as cutoff frequency and quality factor of the 120 Hz filter and the 400 Hz filter, respectively. The bandwidth is determined as

$B_{-3\text{dB},120} = 123$ Hz, $B_{-3\text{dB},400} = 415$ Hz, $B_{\text{ENBW},120} = 137$ Hz and $B_{\text{ENBW},400} = 473$ Hz for the two filter settings, respectively.

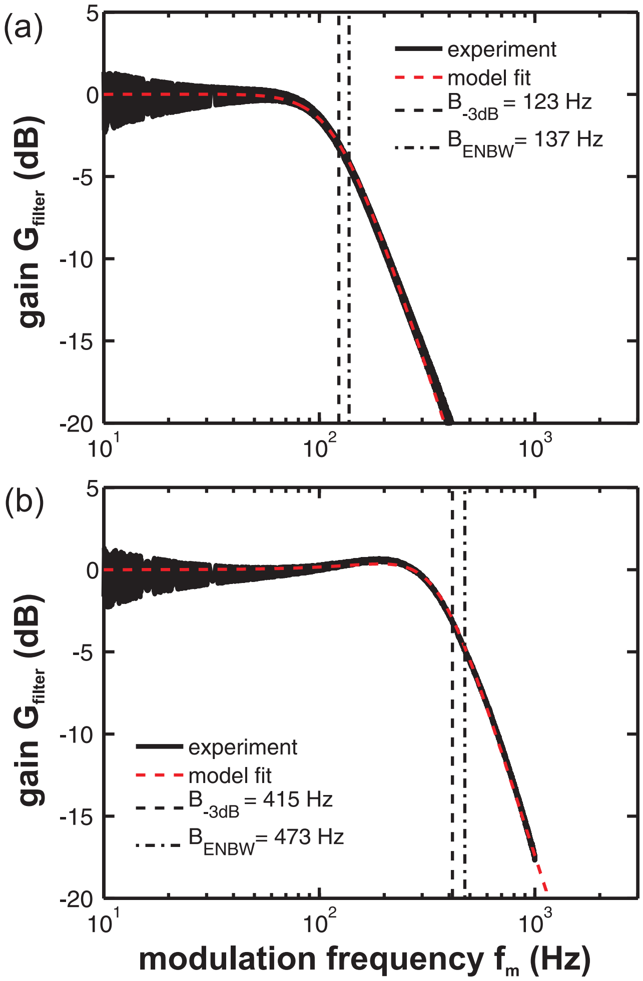

Figure S6: (a) Measured amplitude response of the 120 Hz filter of the easyPLL plus system modelled as a second order low pass. The fit of the model to the experimental data yields $f_c = 120$ Hz and $Q_f = 0.73$. (b) Measured amplitude response of the 400 Hz filter of the easyPLL plus system where the fit yields $f_c = 362$ Hz and $Q_f = 0.83$.

For the **PLLpro2 system**, the transfer function is generally more sophisticated as this system offers a large variety of filter settings. Here, the adjusTable low-pass filter is not located at the Δf output but inside the feedback loop of the PLL. Therefore, the frequency response not only depends on the low-pass filter, but also on the proportional–integral (PI) controller settings and the system has to be described for closed-loop operation [15].

To describe this system in detail, we first recall that the transfer function of the open loop with

the low-pass characteristics H_{LP} and an amplification factor K of the voltage controlled oscillator can be written as [15]

$$H_{\mathrm{OL}}(s) = K \times \frac{H_{\mathrm{LP}}(s)}{s} \quad .$$

The amplification K is split into a proportional part K_P and an integral part K_I. The PI controller determines the response of the feedback system. Its transfer function is inserted into the open-loop function

$$H_{\mathrm{OL}}(s) = K_P \left(1 + \frac{K_I}{s}\right) \times \frac{H_{\mathrm{LP}}(s)}{s} \quad .$$

H_{LP} are Butterworth filters [16] with order o and cutoff frequency f_c. Finally, the transfer function of the closed loop $H_{\mathrm{CL}}(s)$ can be described as [15]

$$H_{\mathrm{CL}}(s) = \frac{H_{\mathrm{OL}}(s)}{1 + H_{\mathrm{OL}}(s)} \quad .$$

The transfer function of the PLLpro2 can hardly be modelled in total due to the complexity of the system. Thus, the amplitude response is measured using the procedure described above. Several curves Δf^m versus f_m are recorded to investigate the influence of the loop filter H_{LP} of order o and cutoff frequency f_c as well as the settings of the PI controller on the frequency response. To relate the settings P and I in the PLL software to the parameters K_P and K_I of our model, a fit of the model to the measured curve is performed for various filter settings and yields prefactors of 369 deg for the P gain and 3.2 for the I gain. These values have to be multiplied to the values of P and I gain, set in the PLL software to obtain a quantitative description of the frequency response. To demonstrate the precision of the predictions, we plot two amplitude response curves in Figure S7. These curves show the amplitude response of the PLL system with $f_c = 1000$ Hz, $n = 3$ and a P gain setting of -2 Hz/deg as a comparison between model and experiment. While we obtain a low-pass behaviour

with varying steepness for an I gain setting of 1 Hz (see Figure S7a), the I gain setting of 200 Hz yields a slight gain peaking at 100 Hz (see Figure S7b).

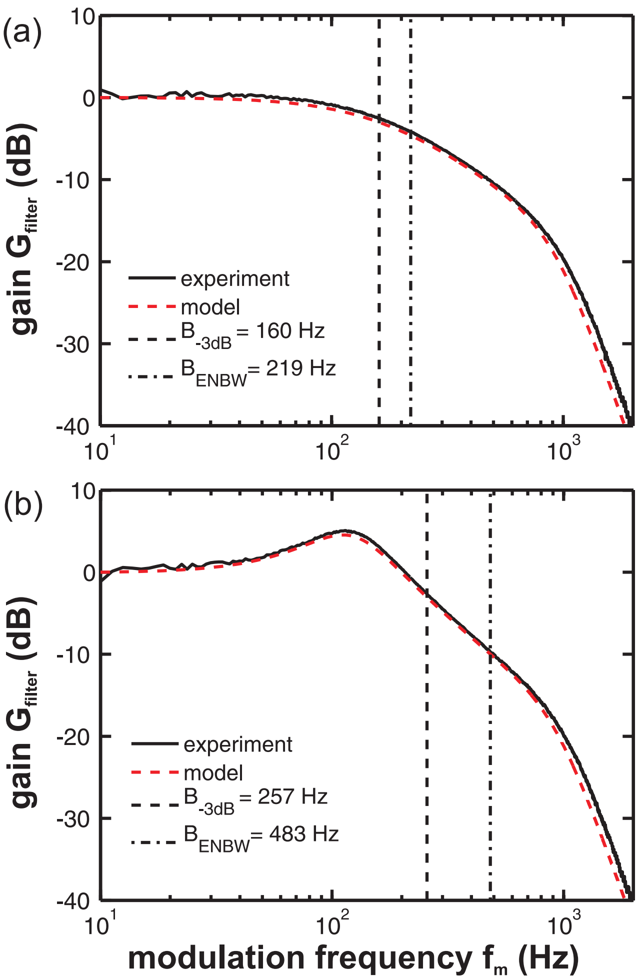

Figure S7: Measured and simulated amplitude response of the PLLpro2 system modelled as a closed loop control. PLL parameters: (a) $f_c = 1000$ Hz, $o = 3$, $K_P = 369\times$ (-2 Hz/deg), $K_I = 3.2\times 1$ Hz. (b) $f_c = 1000$ Hz, $o = 3$, $K_P = 369\times$ (-2 Hz/deg), $K_I = 3.2\times 200$ Hz.

The phase response is calculated as the argument of the transfer function

$$\phi = \arg(H(i\omega)) \quad .$$

Figure S8 shows predictions for amplitude (a) and phase (b) response for different P and I gain settings. These curves demonstrate that there are optimum settings ($P = -2.2$ Hz/deg and $I = 1$ Hz in Figure S8) yielding a rather flat amplitude response over the desired bandwidth and a steep slope

above f_c. If the P and I gain settings are too large, gain peaking occurs. In contrast, a significant attenuation is observed if P is too small. The phase response is also affected by P and I gain settings. Here, the optimum settings yield a smooth response curve with the least pronounced changes in the gradient.

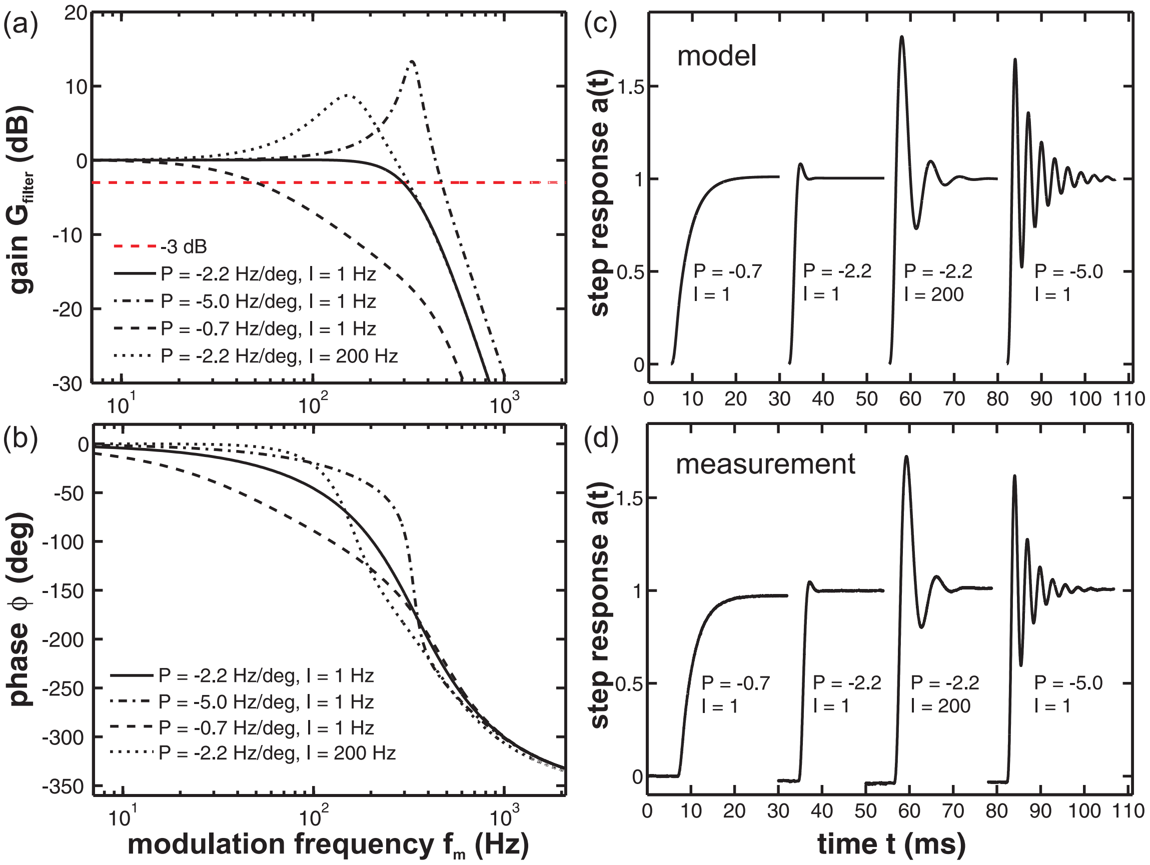

Figure S8: Calculated amplitude response (a) and phase response (b) of the filter of the RHK PLL-pro2 demodulator with $f_c = 500$ Hz, $o = 3$ and different P gain and I gain settings. (c) Step response calculated using Equation 25. (d) Step response measured under the same experimental conditions.

The characteristics of the transfer function are closely related to the step response of the system in the time domain. The step response $a(t)$ can be calculated by applying the inverse Laplace transform

$$a(t) = \mathscr{L}^{-1}\left\{\frac{H_{\mathrm{CL}}(s)}{s}\right\} \tag{25}$$

to the product of the transfer function $H_{\mathrm{CL}}(s)$ and the unit step function $1/s$ [17]. This is performed numerically [18] using a MATLAB script (The MathWorks, Inc., Natick, MA, USA). The respective

graphs are shown in Figure S8c, where the curves demonstrate the result of wrong P and I settings for the step response in terms of overshoot or slow response. As a check for consistency, we directly measure the step response as shown in Figure S8d and find excellent agreement with data from Figure S8c. Note that the optimum for the amplitude and phase response corresponds to the optimum step response ($P = -2.2$ Hz/deg, $I = 1$ Hz).

Obviously, such simulations are not only useful for the noise analysis, but can also be used to find optimum values for the PI controller settings of the PLL system.

Table S4: Optimised settings for the P gain of the RHK PLLpro2 frequency feedback loop yielding a gain peaking smaller than 0.1 dB. The I gain is set to 1 Hz.

f_c (Hz)	o	$B_{-3\mathrm{dB}}$ (Hz)	B_{ENBW} (Hz)	P (Hz/deg)
125	1	103	119	−1.25
125	5	48	57	−0.35
250	1	207	235	−2.51
250	5	96	110	−0.7
500	1	413	467	−5.02
500	2	368	395	−3.14
500	3	305	326	−2.26
500	4	240	264	−1.74
500	5	192	217	−1.40
1000	1	826	929	−10.03
1000	3	609	646	−4.51
1000	5	385	432	−2.81
2000	3	1217	1289	−9.02
2000	5	769	857	−5.61

In Table S4, the ideal P gain values for several combinations of filters and cutoff frequencies are shown to provide a proven set of filter parameters for the PLLpro2 system useful in practice. As a rule of thumb, we find that the higher the bandwidth, the more the P gain has to be reduced. We find that adjusting the P gain is critical, as a wrong setting easily results in gain peaking in the vicinity of f_c. Adjusting the I gain is less critical. High values yield a moderate gain enhancement in a frequency region below f_c. Therefore, we chose a setting of $I = 1$ Hz for most of our measurements and also for the simulations presented here. A typical setting for our scan environment for high-resolution measurements is $f_c = 500$ Hz, $o = 3$, $P = -2.0$ Hz/deg and $I = 1$ Hz.

4 RMS noise

The RMS-noise δf_{tot} in the frequency-shift signal Δf can be obtained from the integration of the Δf noise power spectral density over the entire frequency range

$$\delta f_{\text{tot}} = \left(\int_0^{\infty} D_{\text{tot}}^{\Delta f}(f_m) d f_m \right)^{1/2} \quad . \tag{26}$$

For the analysis of our experiments, we perform a numerical integration of $D_{\text{tot}}^{\Delta f}(f_m)$ from 0 to 10 kHz as the noise power spectral density is always negligible above 10 kHz.

For the case where the spectral function $D_{\text{tot}}^{\Delta f}(f_m)$ is not known, the following approximative expression has been suggested [19] to estimate the RMS noise:

$$\begin{aligned} (\delta f_{\text{tot}}^{\star})^2 &= (\delta f_{\text{th}}^{\star})^2 + (\delta f_{\text{ds}}^{\star})^2 \\ &= \int_0^{B} \widetilde{D}_{\text{tot}}^{\Delta f} d f_m \\ &= \frac{f_0 k_{\text{B}} T}{\pi k_0 Q_0 A^2} B + \frac{2 D_{\text{ds}}^{z}}{3A^2} B^3 \quad . \end{aligned} \tag{27}$$

This expression results from integrating the unfiltered frequency-shift noise $\widetilde{D}_{\text{tot}}^{\Delta f}$ over the bandwidth B. To reveal the discrepancy between the precise result from Equation 26 and the approximation

from Equation 27, we represent δf_{tot}^2 as:

$$\begin{aligned}
\delta f_{\text{tot}}^2 &= \delta f_{\text{th}}^2 + \delta f_{\text{ds}}^2 \\
&= \int_0^\infty D_{\text{th}}^{\Delta f} \, df_m + \int_0^\infty D_{\text{ds}}^{\Delta f} \, df_m \\
&= \int_0^\infty G_{\text{filter}}^2 \widetilde{D}_{\text{th}}^{\Delta f} \, df_m + \int_0^\infty G_{\text{filter}}^2 \widetilde{D}_{\text{ds}}^{\Delta f} \, df_m \\
&= \frac{f_0 k_{\text{B}} T}{\pi k_0 Q_0 A^2} \underbrace{\int_0^\infty G_{\text{filter}}^2 \, df_m}_{B_{\text{ENBW}}} + \frac{2D_{\text{ds}}^z}{A^2} \int_0^\infty G_{\text{filter}}^2 f_m^2 \, df_m \\
&= \frac{f_0 k_{\text{B}} T}{\pi k_0 Q_0 A^2} B_{\text{ENBW}} + \frac{2D_{\text{ds}}^z}{A^2} \int_0^\infty G_{\text{filter}}^2 f_m^2 \, df_m
\end{aligned}$$

where the thermal noise component of the RMS noise δf_{th} can be written in dependence of the equivalent noise bandwidth as defined in Equation 24. The insertion of B_{ENBW} in Equation 27 reveals that these expressions are identical for the thermal noise but not for the detection system noise. Comparing the approximative result of Equation 27 to the precise result of Equation 26 for realistic PLL filter settings, we find that the approximation underestimates the total noise typically by 20% to 30%. A comparison for various filter settings is shown in Table S5.

Table S5: Comparison of precise and approximate determination of the RMS noise. The deviation is calculated as $(\delta f_{\text{ds}} - \delta f_{\text{ds}}^{\star})/\delta f_{\text{ds}}$ according to Equation 26 and Equation 27. The PLL demodulators are modelled as described in detail in Section 3.

PLL system	f_c (Hz)	o	deviation
A	300	2	21.9 %
B	120	2	33.4 %
B	400	2	23.6 %
C	250	1	29.8 %
C	250	5	19.7 %
C	500	1	29.2 %
C	500	2	11.4 %
C	500	3	11.4 %
C	500	4	15.4 %
C	500	5	19.8 %
C	1000	5	19.5 %

References

1. Simon, G. H.; Heyde, M.; Rust, H. P. *Nanotechnology* **2007**, *18*, 255503. doi:10.1088/0957-4484/18/25/255503

2. Giessibl, F. J. *Phys. Rev. B* **1997**, *56*, 16010–16015. doi:10.1103/PhysRevB.56.16010

3. Giessibl, F. J.; Bielefeldt, H. *Phys. Rev. B* **2000**, *61*, 9968–9971. doi:10.1103/PhysRevB.61.9968

4. Nyquist, H. *Phys. Rev.* **1928**, *32*, 110–113.

5. Reif, F. *Fundamentals of statistical and thermal physics*; McGraw-Hill series in fundamentals of physics; McGraw-Hill: Auckland, 2008.

6. Butt, H. J.; Jaschke, M. *Nanotechnology* **1995**, *6*, 1–7. doi:10.1088/0957-4484/6/1/001

7. Melcher, J.; Hu, S. Q.; Raman, A. *Appl. Phys. Lett.* **2007**, *91*, 053101. doi:10.1063/1.2767173

8. Lozano, J. R.; Kiracofe, D.; Melcher, J.; Garcia, R.; Raman, A. *Nanotechnology* **2010**, *21*, 465502. doi:10.1088/0957-4484/21/46/465502

9. Elmer, F. J.; Dreier, M. *J. Appl. Phys.* **1997**, *81*, 7709–7714. doi:10.1063/1.365379

10. Lübbe, J.; Tröger, L.; Torbrügge, S.; Bechstein, R.; Richter, C.; Kühnle, A.; Reichling, M. *Meas. Sci. Technol.* **2010**, *21*, 125501. doi:10.1088/0957-0233/21/12/125501

11. Cook, S.; Schaffer, T. E.; Chynoweth, K. M.; Wigton, M.; Simmonds, R. W.; Lang, K. M. *Nanotechnology* **2006**, *17*, 2135–2145. doi:10.1088/0957-4484/17/9/010

12. Kobayashi, K.; Yamada, H.; Matsushige, K. *Rev. Sci. Instrum.* **2009**, *80*, 043708. doi:10.1063/1.3120913

13. Polesel-Maris, J.; Venegas de la Cerda, M. A.; Martrou, D.; Gauthier, S. *Phys. Rev. B* **2009**, *79*, 235401. doi:10.1103/PhysRevB.79.235401

14. Kittel, P.; Hackleman, W. R.; Donnelly, R. J. *Am. J. Phys.* **1978**, *46*, 94–100. doi:10.1119/1.11171

15. Robins, W. P. *Phase Noise in Signal Sources*; IET Telecommunications Series 9; The Institution of Engineering and Technology: London, 2007.

16. Butterworth, S. *Experimental Wireless & the Wireless Engineer* **1930**, *7*, 536–541.

17. Kauppinen, J.; Partanen, J. *Fourier Transforms in Spectroscopy*; Wiley-VCH: Berlin, 2001.

18. Valsa, J.; Brančik, L. *Int. J. Numer. Model.* **1998**, *11*, 153–166. doi:10.1002/(SICI)1099-1204(199805/06)11:3<153::AID-JNM299>3.0.CO;2-C

19. *Noncontact Atomic Force Microscopy*; Morita, S., Giessibl, F. J., Wiesendanger, R., Eds.; Springer: Berlin, 2009; Vol. 2. doi:10.1007/978-3-642-01495-6_3
